# Pain in multiple sclerosis: clinical phenotypes and therapeutic strategies - a narrative review

**DOI:** 10.1097/PR9.0000000000001403

**Published:** 2026-02-17

**Authors:** Livia Sophie Lang, Maria Protopapa, Stefan Bittner, Frank Birklein

**Affiliations:** Department of Neurology, University Medical Centre of the Johannes Gutenberg University Mainz, Mainz, Germany

**Keywords:** Multiple sclerosis, Pain phenotypes, Neuropathic pain, Nociplastic pain, Nociceptive pain

## Abstract

**Introduction:**

Pain is a frequent and burdensome symptom in multiple sclerosis (MS), yet it remains underrecognized and undertreated.

**Objective:**

This review summarizes current knowledge on the prevalence, mechanisms, clinical phenotypes, and therapeutic strategies of MS-related pain, with an emphasis on implications for clinical management.

**Methods:**

We conducted a narrative review of the literature, integrating epidemiological, clinical, and mechanistic evidence on pain in MS.

**Results:**

Pain is reported by 29% to 86% of people with MS, and up to one-third describe it as their most distressing symptom. Multiple sclerosis–related pain can be classified mechanistically as nociceptive, neuropathic, nociplastic, or mixed. Common clinical syndromes include migraine, tension-type headache, optic neuritis–related pain, trigeminal neuralgia, painful spasticity, and Lhermitte sign. Nociplastic, fibromyalgia-like widespread pain has recently gained attention but remains poorly defined. Evidence for pharmacological interventions - such as anticonvulsants, tricyclic antidepressants, muscle relaxants, corticosteroids for acute optic neuritis, and nabiximols for spasticity - is limited, with few trials addressing specific pain subtypes. Among nonpharmacological strategies, structured exercise shows the most consistent benefits for MS-related pain, whereas evidence for other approaches, including physiotherapy, psychological interventions, and neuromodulation, remains limited. Nevertheless, multimodal strategies addressing co-occurring fatigue, depression, and sleep disturbance appear most effective.

**Conclusion:**

Pain is common and disabling across all MS phenotypes but is often insufficiently addressed in clinical practice. A mechanism-based classification combined with individualized, multimodal treatment offers the most promising framework to improve patient outcomes.

## Abstract 2

Review of pain in multiple sclerosis: heterogeneity of pain phenotypes, underlying mechanisms, and clinical implications for individualized multimodal treatment.

**Key Points**

- Pain is a frequent and disabling symptom in multiple sclerosis, with prevalence estimates ranging from 29% to 86%.
- Mechanistic classification into nociceptive, neuropathic, nociplastic, and mixed pain improves diagnostic and therapeutic precision.
- The most common specific pain syndromes include trigeminal neuralgia, migraine, optic neuritis–related pain, spasticity-associated pain, and neuropathic extremity pain.
- Evidence for pharmacological management is limited; multimodal strategies integrating nonpharmacological interventions are recommended.

## 1. Introduction

Multiple sclerosis (MS) is a chronic immune-mediated disease of the central nervous system characterized by recurrent inflammation, demyelination, axonal injury, and neurodegeneration.^25^ With a prevalence of approximately 2.8 million people worldwide, MS is one of the leading causes of nontraumatic disability in young adults.^44^ The disease manifests in distinct clinical phenotypes: the most common is relapsing–remitting MS, which may transition into a secondary progressive MS course; less frequently, patients present with primary progressive MS. Nowadays, these clinical phenotypes are considered as distinct manifestations of underlying focal and progressive biological processes. Relapses reflect acute inflammatory activity in the brain or spinal cord, producing new neurological symptoms such as motor weakness, sensory loss, imbalance, or visual impairment. By contrast, progression is characterized by gradual worsening of disability without clinical or radiological signs of acute inflammation. This insidious deterioration is strongly associated with brain atrophy. Importantly, progression is not limited to secondary progressive MS but can be observed already from the beginning of the disease.^6^

Pain is increasingly recognized as a frequent and burdensome symptom across all disease courses. Estimates of prevalence range widely between 29% and 86%,^33^ reflecting methodological differences and a lack of standardized definitions. This variability is mirrored in clinical practice: pain is often insufficiently assessed and addressed during routine neurological encounters, despite its high relevance for patients. In fact, up to one third of individuals with MS identify pain as the most distressing symptom of their disease.^43^ Pain is further linked to worse physical disability,^20^ higher rates of fatigue or depression,^17^ impaired work ability, and markedly reduced quality of life.^19,26^ Different types of pain in MS may arise through multiple pathways: as a direct consequence of acute inflammatory lesions during relapses, secondarily because of neurological deficits and immobility, or in the context of progressive pathology.

Despite this high burden, pain remains underrepresented in both research and clinical MS trials. Pivotal studies of disease-modifying therapies have focused primarily on relapse suppression and disability progression, whereas pain outcomes were not systematically recorded. It remains unclear whether modulation of neuroinflammation alters the diverse pain syndromes in MS. In clinical practice, however, this gap is critical: therapeutic strategies differ substantially between pain mechanisms, and an accurate diagnosis is essential to guide both pharmacological and nonpharmacological management.

A further challenge lies in the heterogeneity of pain syndromes in people with MS (pwMS). Patients may experience pain because of musculoskeletal overload, spasticity, or inflammation (eg, optic neuritis); neuropathic pain from demyelinating or axonal lesions in the central somatosensory system (eg, trigeminal neuralgia, burning extremity pain, Lhermitte's sign); or, less well-characterized, generalized pain syndromes, reflecting altered central pain processing. Mixed pain syndromes are common and often overlap with sleep disturbance or mood changes. This complexity underscores the need for a mechanism-based classification of MS-related pain that links clinical phenotypes to underlying pathophysiological processes.

## 2. Rationale for a mechanism-based classification

Different classifications of MS-related pain have been proposed^33,45^ - some descriptive, some mechanism-based - but still no unified framework exists. In this narrative review, we integrate clinical–epidemiological and mechanistic perspectives, with a particular focus on therapeutic implications. It builds on the classification systems proposed by O'Connor et al. (2008) and Truini et al. (2013).^33,45^ Their work highlighted that MS-related pain is not a uniform symptom but a spectrum of syndromes with distinct origins. Clinicians are often confronted with this heterogeneity in daily practice, where pain may arise from inflammatory, neurodegenerative, musculoskeletal, or central sensitization processes. This diversity can make diagnosis and treatment challenging, particularly since pain is frequently underrecognized in neurological routine care.

Mechanistically, the taxonomy introduced by the International Association for the Study of Pain distinguishes nociceptive, neuropathic, and nociplastic pain, as well as mixed forms.^32^ Applying this definition to MS is valuable because it not only provides conceptual clarity but also supports therapeutic decision-making, given that different mechanisms respond to different interventions.

(1) *Nociceptive pain* arises from nonneuronal tissue injury and subsequent activation and sensitization of nociceptors.^39^ In MS, this may present as musculoskeletal back pain or painful spasticity. Headache associated with MS, as well as pain during optic neuritis, also activate nociceptors through inflammation and should be primarily classified as nociceptive.
(2) *Neuropathic pain* results from damage to the somatosensory nervous system, either peripheral or central. It is typically described as burning, stabbing, or shooting, and may be accompanied by allodynia, sensory deficits, or hyperalgesia.^39^ In MS, neuropathic pain in the limbs, which follows a lesion to the spinal cord, and trigeminal neuralgia are the most characteristic examples.
(3) *Nociplastic pain* refers to altered pain perception because of central sensitization in the absence of demonstrable neuronal or nonneuronal tissue damage.^30^ This recently specified pain category is best exemplified by fibromyalgia. In MS, prevalence and clinical features remain poorly defined, with only one observational study suggesting a potential contribution.^31^
(4) *Mixed pain syndromes* are common in MS, patients frequently present with overlapping pain types. An example would be painful spasticity co-occurring with burning neuropathic extremity pain.

**Figure 1.**
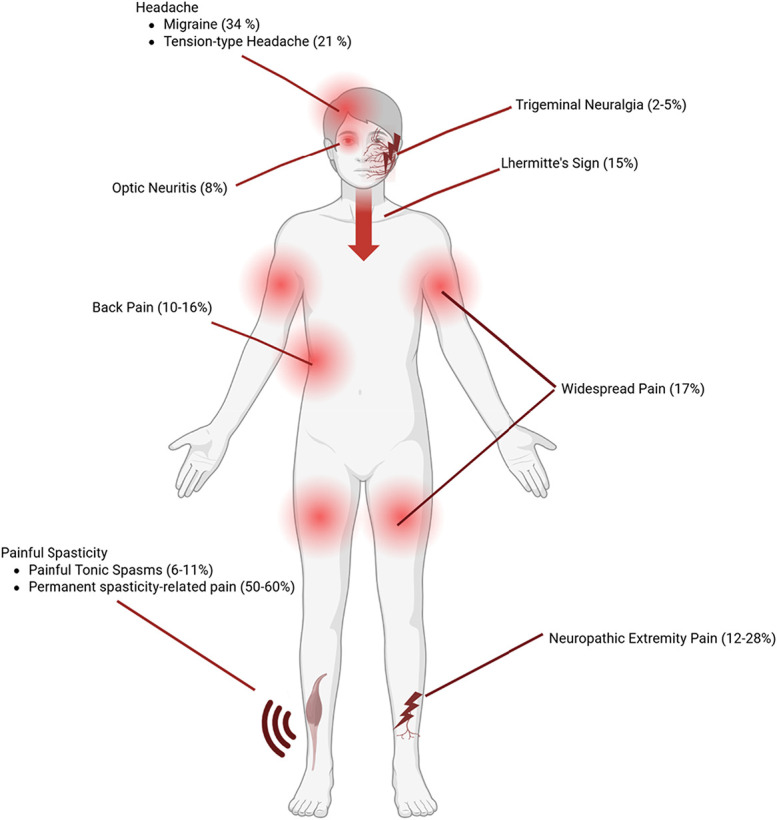
Graphical representation of common pain syndromes observed in multiple sclerosis. Prevalence estimates (in parentheses) are based on Truini et al. Created with BioRender.com.

In addition to mechanism-based approaches, MS-related pain can also be classified according to the anatomical localization. Clinically, it might be useful to distinguish between head and facial pain vs body and extremity pain. Figure 1 illustrates the most common pain types in MS, stratified by localization and estimated prevalence.

## 3. Clinical spectrum of pain in multiple sclerosis

In the following sections, we describe the most frequent pain syndromes in MS, highlighting their clinical characteristics, underlying mechanisms, and available treatment options (an overview of pharmacological strategies is provided in Table 1).

**Table 1:** Overview of different pharmacological approaches.

Type of pain	Pharmacological therapy
Neuropathic pain	
Trigeminal neuralgia	Carbamazepine, oxcarbazepine, phenytoin; alternatively lamotrigine, lacosamide, gabapentin
Neuropathic extremity pain	Pregabalin, gabapentin, amitriptyline, duloxetine
Lhermitte's sign	If necessary: carbamazepine
Nociceptive pain	
Tension-type headache	Acute treatment: NSAIDs Prophylaxis: amitriptyline
Migraine	Acute treatment: NSAIDs, triptans, CGRP receptor antagonists Prophylaxis: beta-blockers, calcium channel blockers, amitriptyline, monoclonal antibodies targeting CGRP/CGRP receptors
Optic neuritis-related pain	High-dose corticosteroids as part of acute MS treatment; in rare cases of persistent pain, long-acting NSAIDs
Back pain	NSAIDs and co-analgesics; opioids in selected cases
Painful tonic spasms	Baclofen, tizanidine, cannabinoids as add-on; in special cases gabapentin, carbamazepine, or lacosamide may be useful
Permanent spasticity-related pain	Baclofen, tizanidine, gabapentin, cannabinoids as add-on
Nociplastic pain	
Widespread fibromyalgia-like pain	Amitriptyline; pregabalin (with comorbid anxiety); duloxetine (with comorbid depression)
Others	
Therapy-associated pain	Symptomatic treatment (NSAIDs for myalgia/headache; pregabalin, gabapentin, amitriptyline for neuropathic complaints) or switch of immunomodulatory therapy

CGRP, calcitonin gene-related peptide; MS, multiple sclerosis; NSAIDs, nonsteroidal anti-inflammatory drugs.

### 3.1. Neuropathic facial pain

#### 3.1.1. Trigeminal neuralgia

Trigeminal neuralgia (TN) is characterized by paroxysmal, lancinating, electric shock–like pain attacks in the distribution of the trigeminal nerve. Attacks may occur spontaneously or be triggered by innocuous stimuli such as eating, speaking, or touch, and typically last only a few seconds. In MS, TN arises from demonstrable damage or irritation of trigeminal afferents, most often because of demyelinating plaques at the root entry zone in the pons, which can often be visualized in magnetic resonance imaging. Compared with the general population, pwMS have a roughly 20-fold increased risk of developing TN^12^ and bilateral cases are substantially more frequent.^23^ Only in a minority of patients, a vascular compression contributes to the pathophysiology, which may have therapeutic implications.^46^

### 3.2. Nociceptive head and facial pain

#### 3.2.1. Tension-type headache

Tension-type headache (TTH) is common in the general population but does not appear to be increased in prevalence among pwMS. It is typically bifrontal, occipital, or holocephalic and, unlike migraine, it often improves with physical activity.

#### 3.2.2. Migraine

The prevalence of several headache types is higher in pwMS compared with the general population, with migraine being the most prominent. Beyond increased prevalence, a temporal association between migraine attacks and MS relapses has been discussed.^33^ Although lesion localization within the central nervous system may contribute, the underlying mechanisms remain incompletely understood. Migraine typically presents with recurrent, unilateral, pulsating headaches, lasting up to 72 hours and frequently accompanied by nausea, photophobia, and phonophobia. Migraine with aura involves transient neurological deficits such as visual disturbances (eg, fortifications), sensory symptoms, or transient paresis. The pathophysiology involves spreading depression, release of vasodilatory neuropeptides, and activation of nociceptors.^8^

#### 3.2.3. Optic neuritis-related pain

Optic neuritis (ON) represents a frequent cause of acute pain in MS. In earlier prevalence studies,^24^ ON-related pain was often excluded because of the lack of standardized diagnostic criteria at the time. Although ON-related pain occurs frequently over the course of the disease, its point prevalence is relatively low, as symptoms are usually transient and tend to resolve after the acute episode. Pain arises from inflammation of the optic nerve and surrounding structures (eg, nervi nervorum, optic sheath), producing retrobulbar pain particularly aggravated by eye movements.^45^ Optic neuritis is typically associated with vision loss and impaired color perception in the affected eye.

### 3.3. Neuropathic pain of the body and extremities

#### 3.3.1. Neuropathic extremity pain

Many pwMS experience persistent neuropathic pain affecting the trunk or limbs. The pain is often described as burning, stabbing, or pulling, typically with a distal emphasis that may be unilateral or bilateral, and related to a spinal lesion. Sensory disturbances are usually present in the painful area. Although the precise mechanisms are unclear, lesions of the spinothalamic tract or impaired descending pain control have to be considered.^42^

#### 3.3.2. Lhermitte´s sign

Lhermitte´s sign refers to electric-shock-like sensations radiating down the spine and into the limbs upon neck flexion. The phenomenon is transient, usually lasting only seconds and disappears when the head is returned to a neutral position.^2^ It is attributed to cervical demyelinating lesions and to heightened excitability of the affected axons, particularly when stretched.^2^

### 3.4. Nociceptive pain of the body and extremities

#### 3.4.1. Back pain

Back pain is more common in people with MS than in the general population. Although the contribution of MS-specific mechanisms remains unclear, abnormal posture because of paresis or spasticity and impaired mobility are considered as major drivers, leading to musculoskeletal overload and secondary pain.^33^

#### 3.4.2. Painful spasticity

Spasticity is a frequent feature in the course of MS and can itself become a major source of pain. Two distinct forms can be differentiated, which differ in etiology, clinical presentation, and therapeutic implications. Yet, in pharmacological trials investigating antispastic agents, the presence or absence of pain is rarely considered as an outcome, limiting our understanding of treatment effects on painful spasticity. Independent of type, spasticity severity should be assessed regularly, for example, using a numerical rating scale.^15^

##### 3.4.2.1. Painful tonic spasms

These brief, stereotyped, involuntary muscle contractions are highly characteristic of MS. They can occur unilaterally or bilaterally and typically last <2 minutes. Clinically, 4 subtypes have been distinguished: flexor spasms, extensor spasms, adductor spasms, and trunk/back spasms. The underlying mechanism is thought to involve hyperexcitability of alpha-motoneurons because of MS lesions in the central nervous system. Simultaneous activation of adjacent motor units produces considerable mechanical stress, activating nociceptors and thereby eliciting pain. Truini et al. further proposed that compression of intramuscular blood vessels during spasms could add an ischemic pain component.^45^

##### 3.4.2.2. Permanent spasticity-related pain

By contrast, permanent spasticity-related pain is defined by a persistently elevated muscle tone, with exacerbations triggered by specific movements (often of the hip adductors) that typically last longer than 2 minutes.^45^ Its pathophysiological basis differs from tonic spasms: presynaptic disinhibition leads to an exaggerated stretch reflex, whereas reflex-driven motoneuron activity prevents coordinated contraction of adjacent motor units. Pain is believed to result from microscopic muscle injury and the release of pronociceptive mediators during stretching of contracted muscle fibers or tendons.^45^

### 3.5. Nociplastic pain

Nociplastic pain is not included in most established classification systems of MS-related pain, but a small number of studies have addressed it as a potentially relevant component.^9,20^ It is characterized by altered central pain processing in the absence of structural nerve damage and resembles the pain phenotype observed in fibromyalgia. Patients typically describe diffuse, muscle soreness–like pain affecting large body regions (widespread), often accompanied by fatigue, sleep disturbance, and mood changes.

The first study specifically examining this pain entity in MS estimated a prevalence of around 17%.^9^ More recent work reported that approximately one-quarter of pwMS who experience chronic pain fulfill criteria^47^ for nociplastic pain, with an additional subgroup exhibiting mixed neuropathic–nociplastic feature.^31^ These patients tend to report higher pain intensity, multiple coexisting pain conditions, and greater impairment in quality of life. Findings from a large US survey further confirmed the clinical relevance of widespread nociplastic pain in MS, linking it to reduced physical activity and poorer overall functioning.^1^

### 3.6. Therapy-associated pain

Pain in MS can also occur as a side effect of disease-modifying therapies. Interferon-beta frequently induces flu-like symptoms such as headache, myalgia, and abdominal pain and is often accompanied by injection-site reactions, also seen with glatiramer acetate. By contrast, injection-site pain is less frequent with subcutaneous anti-CD20 treatment (e.g. ofatumumab).^37^ Teriflunomide has been associated with the development of painful peripheral polyneuropathy, whereas repeated corticosteroid use increases the risk of osteoporosis and fracture-related pain.^37^

For structured clinical evaluation across different pain phenotypes, the use of standardized instruments and regular assessment of pain intensity with the numerical rating scale is recommended. However, no pain questionnaire specifically validated for MS is currently available.

## 4. Therapeutic approaches

Given the heterogeneity of MS-related pain syndromes, therapeutic strategies must be tailored to the underlying mechanisms. In clinical practice, pharmacological treatment remains the cornerstone, but the evidence base is limited by small trials, heterogeneous outcome measures, and the frequent neglect of pain as an endpoint in MS studies. When prescribing analgesics or co-analgesics, clinicians should be aware that many agents may aggravate fatigue or cognitive slowing - symptoms already common in MS - and careful dose titration and monitoring are therefore essential. Current recommendations largely draw on national guidelines, with the German MS guideline being the most up to date and comprehensive in addressing pain.^21^ In addition, patient-centered resources such as the German MS quality manual have been considered because they reflect both clinical standards and real-world applicability. Below, we summarize available therapeutic options structured by pain phenotype, followed by an overview of nonpharmacological and multimodal approaches. An overview of pharmacological agents is provided in Table 1.

### 4.1. Pharmacological strategies

#### 4.1.1. Neuropathic pain

*Trigeminal neuralgia* (TN) in MS is managed according to the same principles as in primary TN. First-line therapy consists of sodium channel blockers such as carbamazepine or oxcarbazepine, with phenytoin, lamotrigine, or lacosamide representing additional options. However, it is important to recognize that pwMS may have a reduced therapeutic window for these agents: adverse effects can occur at lower dosages, and neurological side effects may be misinterpreted as relapse activity. Recent evidence also supports gabapentin as effective and potentially safer alternatives for patients who do not tolerate or respond to sodium channel blockers.^50^

Invasive procedures should only be considered in MS patients after exhaustion of conservative measures. Among these, radiosurgical approaches such as Gamma Knife therapy are particularly recommended, offering a minimally invasive option with favorable efficacy and safety.^41^ Percutaneous procedures (eg, balloon compression or radiofrequency thermocoagulation) remain established alternatives, whereas microvascular decompression should be reserved for selected patients with demonstrable neurovascular compression.^46^

*Neuropathic extremity pain* is typically treated in line with general neuropathic pain guidelines. Tricyclic antidepressants, gabapentin, or pregabalin are considered first-line agents. Preliminary studies suggest a potential benefit of cannabinoids, although the available evidence is limited and does not yet allow firm recommendations.^16^

*Lhermitte´s sign* is usually self-limiting, occurring in the context of acute relapses and resolving within weeks. In persistent cases, despite relapse treatment, a treatment attempt with a sodium channel blocker such as carbamazepine may be considered, based on its ability to reduce neuronal hyperexcitability.

#### 4.1.2. Nociceptive pain

*Tension-type headache* (TTH) in MS is treated analogously to idiopathic TTH. Nonsteroidal anti-inflammatory drugs (NSAIDs) are used for acute attacks. In cases of chronic TTH, defined as occurring on >15 days per month for at least 3 months, prophylactic treatment with amitriptyline or duloxetine is recommended.

*Migraine* therapy does not differ between people with and without MS. Evidence-based acute treatment includes NSAIDs, triptans, and calcitonin gene-related peptide (CGRP) receptor antagonists. For patients with frequent migraine attacks, prophylactic options comprise beta-blockers (metoprolol, propranolol), calcium antagonists (flunarizine), amitriptyline, or monoclonal antibodies targeting CGRP or its receptor. Valproic acid and topiramate may also be considered in men or when conception is prohibited in women.^13^ Given the susceptibility to central side effects in MS, CGRP-targeted agents may be particularly useful when conventional prophylactics are poorly tolerated. When combining beta-blockers with sphingosine-1-phosphate (S1P) modulators, the additive bradycardic effects of both drugs must be carefully monitored.^37^

*Optic neuritis (ON)–*related pain is typically managed with high-dose corticosteroids, although there is no evidence of a sustained long-term benefit. In clinical practice, corticosteroids are widely used to accelerate recovery from acute inflammation.^18^ In rare cases of persistent ON-related pain, treatment with a long-acting NSAID such as etoricoxib may be considered.

*Back pain* in MS can be approached according to general guidelines for chronic nonspecific low back pain. Nonopioid analgesics and co-analgesics such as amitriptyline or gabapentin/pregabalin are first-line options. In selected cases, short-term use of weak opioids may be considered under close supervision, although national guidelines differ in their recommendations and emphasize strict re-evaluation and avoidance of opioid therapy.^7^ Importantly re-evaluation is required after 3 months, and if no meaningful reduction in pain is achieved, opioid therapy should be discontinued. A recent phase 3 trial in chronic low-back pain reported small but significant analgesic effects of a balanced oral tetrahydrocannabinol (THC)/cannabidiol (CBD) extract, indicating potential benefit for low back pain, particularly when spasticity contributes (see painful spasticity).^27^

*Painful spasticity* remains a therapeutic challenge, as available evidence is limited by small study populations and insufficient differentiation between spasticity subtypes.^34^ According to current guidelines, treatment begins with oral muscle relaxants such as baclofen or tizanidine,^21^ which require careful titration and monitoring of treatment response, as low doses may be insufficiently effective, whereas higher doses can aggravate fatigue or cognitive slowing in people with MS. Clonidine may be considered in selected cases refractory to first-line oral agents.^34^ However, in painful tonic spasms, a therapeutic trial with carbamazepine or lacosamide—both of which reduce neuronal hyperexcitability—may be considered.^36^ Gabapentinoids act at both central and peripheral levels, targeting nociceptive as well as neuropathic mechanisms, and might be therefore useful in permanent spasticity-related pain. There is a growing body of evidence on the use of cannabis-based medicines for pain and spasticity in MS.^4,16^ Nabiximols is approved in several European countries, as an oromucosal spray for add-on therapy of MS-related spasticity. On a special note, in the clinical trials that led to its approval, cannabinoid-based therapies demonstrated benefits for spasticity-related pain, but not for other pain types such as neuropathic pain.^16^ In refractory cases, botulinum toxin A may be used for focal spasticity, whereas intrathecal baclofen pumps represent an option for generalized, treatment-resistant flexor or adductor spasticity of the legs and, in rare cases of severe or functionally limiting focal spasticity, surgical procedures such as tendonotomy or selective neurotomy may be considered.^34^

In addition to pharmacological therapy, early physiotherapy is strongly recommended for patients with MS suffering from back pain or painful spasticity because it can help improve mobility, prevent secondary complications, and support long-term pain management.

#### 4.1.3. Nociplastic pain

As this pain phenotype has been scarcely characterized in MS, no evidence-based treatment recommendations or controlled trials are available. Given its clinical resemblance to fibromyalgia, a pragmatic approach may include physiotherapy and psychoeducation (see nonpharmacological strategies), alongside an empirical trial of pharmacological agents such as amitriptyline, pregabalin (particularly in the presence of comorbid generalized anxiety disorder), or duloxetine (in patients with comorbid depression). Pharmacological management should avoid NSAIDs and especially opioid therapy, as efficacy in nociplastic pain is poor and risks of central sensitization and cognitive side effects are increased.^29^

#### 4.1.4. Therapy-associated pain

Although often reversible, therapy-associated pain can mimic MS-related syndromes and thereby complicate attribution. Depending on severity, management may include symptomatic treatment (eg, NSAIDs for myalgia or headache; pregabalin, gabapentin, or amitriptyline for neuropathic complaints) or, in selected cases, a switch of immunomodulatory therapy. In patients receiving repeated courses of glucocorticoids, clinicians should be aware of the increased risk of osteoporosis and consequent vertebral or other fragility fractures.^5^ Moreover, sensory deficits because of spinal cord lesions may blunt pain perception, potentially delaying recognition.

### 4.2. Nonpharmacological strategies

As in other chronic pain conditions, a multimodal strategy, combining pharmacological and nonpharmacological therapies tailored to individual mechanisms and patient needs, appears most effective in pwMS. Such approaches not only target pain intensity but also address comorbid fatigue, sleep disturbance, and mood symptoms, thereby improving overall quality of life. The following sections summarize the main nonpharmacological strategies for pain management in MS, based on the most recent Cochrane review and international guidelines^3,14,22^ while integrating recent systematic reviews and clinical data for an updated synthesis.

#### 4.2.1. Exercise and Physiotherapeutic/Ergotherapeutic interventions

Structured exercise provides small-to-moderate benefits for MS-related pain and further improves fatigue, mood, and function Physical activity seems beneficial not only for nociceptive but also for neuropathic and nociplastic pain, for which pharmacological options are limited.^1,11,49^ Aerobic and strengthening programs (eg, cycling, walking, swimming) performed at moderate intensity for 30 to 60 minutes, 2 to 3 times weekly, adjusted to disability level, appear most effective and should be recommended to all pwMS with pain.^11^

#### 4.2.2. Psychological approaches

Evidence for psychological interventions (eg, cognitive-behavioural therapy, self-management, hypnosis, electroencephalography (EEG)-based biofeedback) remains limited. Although effects on pain are inconsistent, psychoeducational and coping-oriented programs can improve self-efficacy and psychological well-being and are particularly useful for patients with maladaptive coping or distress.^10^

#### 4.2.3. Neuromodulatory interventions

Techniques such as transcutaneous electrical nerve stimulation (TENS) and transcranial direct-current stimulation aim to modulate neural activity and have shown moderate effects on pain and related symptoms in small studies, although overall evidence quality remains low. Given their safety and accessibility - particularly TENS, which can be self-administered at home - these methods may be considered as adjuncts within a multimodal pain management plan.^38,48^

#### 4.2.4. Mind–body approaches

Yoga, mindfulness, relaxation, biofeedback, hypnosis, and reflexology have mainly been studied for fatigue, mood, and quality of life. Evidence for analgesic effects is weak, but these interventions may complement multidisciplinary care by supporting self-management and psychological well-being. Small individual trials reported improved mood or fatigue, but results were heterogeneous and often underpowered.^28^

#### 4.2.5. Dietary and other complementary interventions

Among dietary and complementary options, only vitamin D has been consistently evaluated regarding its potential disease-modifying effects. However, pain outcomes were not systematically assessed, and its impact on pain therefore remains unknown.^40^ Evidence from small and heterogeneous trials on acupuncture is insufficient to support efficacy. Overall, such approaches may be used as adjuncts when compatible with comedication and patient preference but cannot be recommended as evidence-based pain treatments.^35^

## 5. Summary

Pain is a frequent symptom in people with MS and can substantially impair quality of life. Nevertheless, it is often insufficiently assessed and inadequately treated in clinical practice, partly because of heterogeneous study data and the lack of standardized assessment methods. The most common pain syndromes include migraine, spasticity-related pain, and neuropathic pain. A mechanistic classification into nociceptive, neuropathic, nociplastic, and mixed types is clinically useful because therapeutic approaches differ accordingly. As in other chronic pain conditions, a multimodal treatment strategy that integrates pharmacological and nonpharmacological interventions is particularly recommended.

## Disclosures

The authors have no conflict of interest to declare.
